# Contribution of Cell Surface Hydrophobicity in the Resistance of *Staphylococcus aureus* against Antimicrobial Agents

**DOI:** 10.1155/2016/1091290

**Published:** 2016-02-07

**Authors:** Puja Lather, A. K. Mohanty, Pankaj Jha, Anita Kumari Garsa

**Affiliations:** ^1^Animal Biochemistry Division, National Dairy Research Institute, Karnal, Haryana 132001, India; ^2^Animal Biotechnology Division, National Dairy Research Institute, Karnal, Haryana 132001, India; ^3^Dairy Cattle Nutrition Division, National Dairy Research Institute, Karnal, Haryana 132001, India

## Abstract

*Staphylococcus aureus* is found in a wide variety of habitats, including human skin, where many strains are commensals that may be clinically significant or contaminants of food. To determine the physiological characteristics of resistant strain of* Staphylococcus aureus* against pediocin, a class IIa bacteriocin, a resistant strain was compared with wild type in order to investigate the contribution of hydrophobicity to this resistance. Additional clumping of resistant strain relative to wild type in light microscopy was considered as an elementary evidence of resistance attainment. A delay in log phase attainment was observed in resistant strain compared to the wild type strain. A significant increase in cell surface hydrophobicity was detected for resistant strain in both hexadecane and xylene indicating the contribution of cell surface hydrophobicity as adaptive reaction against antimicrobial agents.

## 1. Introduction

In spite of significant advances in food science and technology, food borne illness and economic losses due to food spoilage are still major concerns in food industry.* S. aureus* is found in a wide variety of habitats, including human skin where many strains are commensals that may be clinically significant or contaminants of food [1]. Staphylococcal food poisoning results from consumption of one or more enterotoxins resulting in symptoms of intoxication. Staphylococcal enterotoxins (SEs) are heat stable enterotoxins by heating [2]. Illness results when preformed toxins in the food are eaten at high enough levels, due to significant growth of* S. aureus* in them.

The continuous use of antibiotics has resulted in multiresistant bacterial strains all over the world [3]. Consequently, there is an urgent need to search for alternatives to synthetic antibiotics. The discovery of diverse population of nontoxic, nonimmunogenic, and potent selective antimicrobial peptides (AMPs), as essential components of anti-infective defense mechanisms in mammals, amphibians, insects, plants, and bacteria, offers effective candidates against bacteria, fungi, viruses, and protozoa that become resistant to synthetic drugs [4, 5]. Regardless of their wide spectrum of effectiveness they possess some common features. They are short peptides with 12–50 amino acids; most of them are cationic in nature and they fold into an amphipathic three-dimensional structures [6]. Bacteriocins are ribosomally synthesized AMPs or proteins that are quite different from the classical peptide antibiotics, which are made through enzymatic condensation of free amino acids [7]. Bacteriocins kill their target by causing dissipation of Proton Motive Force (PMF) and leakage of small intracellular substances through pore formation in the cell membrane of sensitive bacteria [8, 9]. There has been a resurgence of interest for research on bacteriocins in the last decade. Bacteriocins can be used as natural biopreservatives because they are nontoxic as they are inactivated by human digestive tract proteases. Bacteriocins from lactic acid bacteria (LAB) are cationic, amphiphilic molecules composed of 20 to 60 amino acid residues [10]. These are commonly classified into three groups [11]. Lantibiotics (lanthionine-containing bacteriocins) are small (<5 kDa) peptides containing the unusual amino acids lanthionine (Lan), *α*-methyllanthionine (MeLan), dehydroalanine, and dehydrobutyrine. These bacteriocins are grouped as class I. Small (<10 kDa), heat-stable, non-lanthionine-containing peptides are class II bacteriocins. These peptides are divided in two subgroups. Class IIa includes pediocin-like peptides having an N-terminal consensus sequence, Tyr-Gly-Asn-Gly-Val (YGNGV). Class IIb contains bacteriocins requiring two different peptides for activity. The class III bacteriocins are not well characterized. This group includes large (>30 kDa) heat-labile proteins. The class IIa bacteriocins are composed of 37 to 48 residues and have a characteristic YGNGV/L consensus sequence. The development of resistance against bacteriocins threatens their safe use as biopreservatives. The available reports suggest that there are changes in the membrane phospholipid composition from sensitive to resistant varieties against barrel-stave pore-forming AMPs [12–15]. The understanding of mechanism of resistance development will help better understanding of structure of the target bacterial cell envelope and activity of bacteriocin.

## 2. Material and Methods

### 2.1. Bacterial Cultures, Growth Media, and Culture Conditions

*Staphylococcus aureus *NCDC 133 and* Pediococcus pentosaceus *NCDC 273 were procured from National Collection of Dairy Cultures (NCDC), National Dairy Research Institute (NDRI), Karnal, Haryana, India, in freeze-dried form.* S. aureus* was grown in nutrient broth and* P. pentosaceus* was grown in deMan-Rogosa-Sharpe (MRS) broth, HiMedia Laboratories, India.

### 2.2. Pediocin Production and Purification

The antibacterial activity of* P. pentosaceus* NCDC 273 was assessed by the deferred agar spot assay and the spot-on-lawn assay [16]. The* pedA* gene encoding pediocin was detected using PCR and sequenced [17]. Pediocin produced was purified by three-step purification procedure, which included ammonium sulfate precipitation, cation-exchange chromatography, and reverse-phase high-performance liquid chromatography [15]. The purity and antimicrobial activity of the pediocin fraction were checked using SDS-PAGE [18]. Gel was removed and cut into two parts. One half, containing molecular weight marker and the purified pediocin, was stained with CBB R-250. The other half, containing the purified bacteriocin, was overlaid with* Staphylococcus aureus* and incubated at 37°C for 16 h.

### 2.3. Morphology of Wild Type and Resistant Strain of* S. aureus*

The morphological features of both the wild type and resistant strain were studied using light microscopy. Approximately 10 *μ*L or a loopful of cultured bacterial cells was placed on glass slide. Bacterial cells were then heat-fixed at a low heat using a spirit lamp. Then a drop of crystal violet stain was placed on heat-fixed cells and left for 1 min. Then the slides were washed under tap water for 1 min. The slides were dried at room temperature or in oven/incubator. Then the slides were examined under light microscope (1000x). The photographs were taken using digital camera.

### 2.4. Determination of Growth Curves for Wild Type and Resistant Strain

Briefly, in two different tubes of 5 mL sterile nutrient broth, 1% of overnight grown cultures of wild type and resistant variant were added, respectively, and incubated at 37°C till the end of experiment. Optical densities of the cultures were recorded at 600 nm from 0 to 16 h with 2 h interval using fresh media as blank after vortex for 1-2 seconds.

### 2.5. Cell Surface Hydrophobicity

The bacterial cell hydrophobicity of both the wild type and resistant strain of* S. aureus* was determined using MATH (Microbial Adhesion to Hydrocarbons) assay. The cell surface hydrophobicity of both wild type and resistant variants of* S. aureus* was assayed by the MATH/BATH (Microbial Adhesion to Hydrocarbons/Bacterial Adherence to Hydrocarbons) assay as described by Reifsteck et al., [20] with slight modifications. Both wild type and resistant strains of* S. aureus* were cultured in 5 mL of nutrient broth (pH 7.0) to stationary phase at 30°C. The cells were harvested by centrifugation at 3,000 g for 15 min, washed three times in ice-cold phosphate buffer, and finally resuspended in phosphate buffer to achieve an OD_500_ of 0.5. A 4.8 mL volume of each bacterial suspension was mixed with 0.8 mL of* n*-hexadecane in a glass tube and vigorously shaken for 1 min. After 30–60 min, the aqueous phase was carefully removed with a micropipette, and absorbance was determined at 500 nm, using a UV-Visible spectrophotometer. The affinity of bacteria for the solvent, that is, hexadecane and xylene, was evaluated by the following formula: % adherence = (1 − *A*/*A*_0_) × 100, where *A*_0_ is the OD_500_ of the bacterial suspension before mixing and *A* is the OD after mixing with solvent.

## 3. Result and Discussion

**Figure 1:**
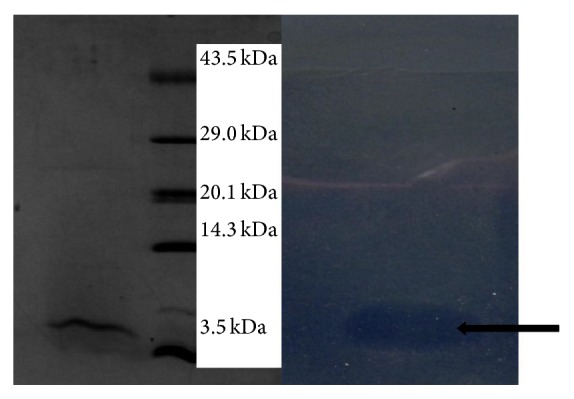
Purified fused pediocin analyzed by SDS-PAGE. Gel was removed and cut into two parts. One half, containing molecular weight marker (lane 2) and the purified pediocin, was stained with CBB R-250. The other half, containing the purified bacteriocin (lane 3), was overlaid with* Staphylococcus aureus* and incubated at 37°C for 16 h. Arrow indicates bacteriocin activity.

**Figure 2:**
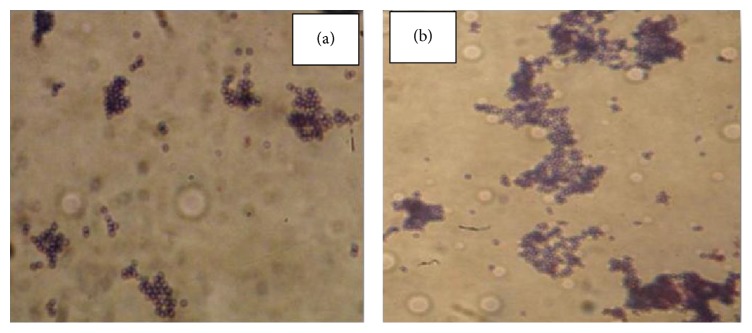
Morphology determination by means of Gram staining examined under light microscope (1000x) of* S. aureus* variants: (a) wild type; (b) resistant strain.

**Figure 3:**
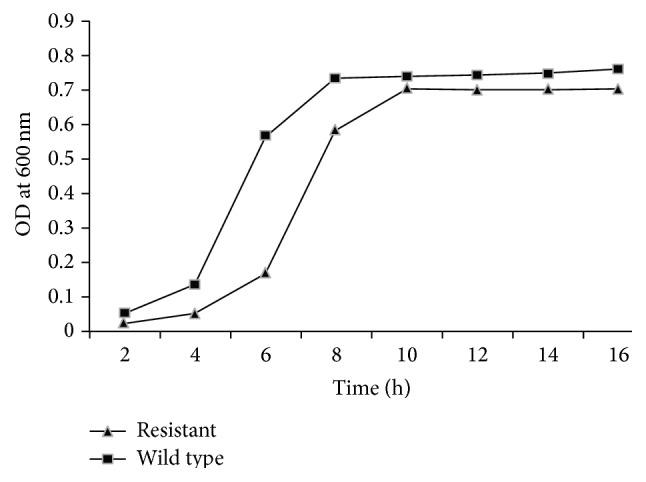
Comparison of growth curve of wild type and resistant* S. aureus* hydrophobicity may be responsible as it leads to enhanced clumping and hence provides less surface area to the microbial colony to expand.

**Figure 4:**
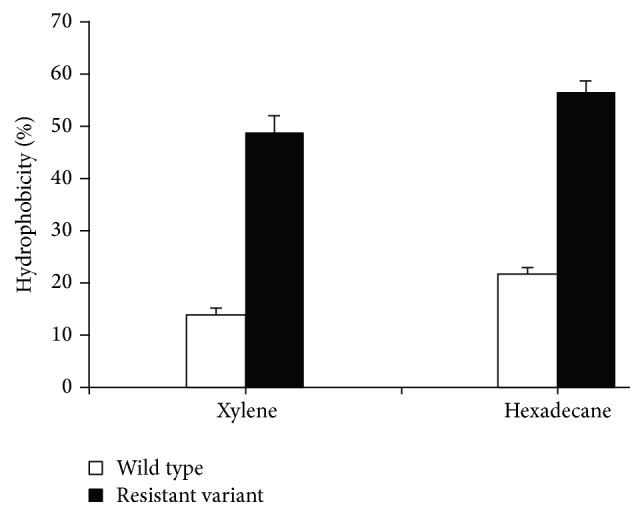
Comparison of percent hydrophobicity of wild type and resistant strain of* S. aureus*. Values are mean ± SD of three independent determinations (*n* = 3).

Barrel-stave pore-forming, class IIa bacteriocins produced by lactic acid bacteria have been widely studied and considered as safe and natural food preservatives [21]. The emergence and spread of resistance against known bacteriocins in food spoilage and pathogenic bacteria would threaten the safety of using bacteriocins as food preservatives [19]. The target bacteria adopt various strategies to overcome the effect of antimicrobial agents by either altering the cell envelope composition which no more remains a suitable target for AMPs or forming bacterial cell clumps by aggregation of large number of bacteria [15, 22, 23].* S. aureus *is highly resistant to antimicrobial factors of the innate immune system such as cationic antimicrobial peptides (CAMPs) [14], which are produced by epithelial cells and neutrophils [24, 25]. It has been reported that food poisoning causing organisms like* S. aureus* and* Listeria monocytogenes* are developing resistance to antimicrobial peptide, nisin [23].* S. aureus* also acquired resistance to platelet microbial protein, a small cationic peptide that possesses potent microbicidal activities against common blood stream pathogens [26].* S. aureus* resistant mutants have been shown to resist Defensins and Protegrins, innate immunity antimicrobial cationic peptides [27]. First pediocin was purified and its confirmation was done using SDS-PAGE. The purified fraction was run on SDS-PAGE; a single band with a molecular mass of 5.0 kDa was observed showing antimicrobial activity against* S. aureus* which confirmed the presence of pediocin (Figure 1). Inhibitory activity of pediocin was also observed earlier against* S. aureus* [28]. Both wild type and resistant variant of* S. aureus* were selected on basis of IC_50_ (Table 1) [19]. Selection of resistant mutant was a stable phenotype as it remained distinguishing characteristic even in absence of pediocin [12]. After Gram staining small bunches of cells were observed for wild type while larger clumps were observed in case of pediocin resistant* S. aureus *(Figure 2). The results revealed more clumping in resistant strain, which indicates that the bacteria acquire a protective shield which leads to lesser exposure to surroundings and as a result bacteriocin could interact only with the outer layer of bacteria leaving the inner layers of bacteria alive [15]. A delay in the start of log phase was observed on development of resistance against pediocin by* S. aureus*. The lag phase increased from 2 h in case of wild type strain to nearly 4 h in case of resistant strain (Figure 3). The log phase duration increased in case of resistant bacteria and there was a delay in attainment of stationary phase. Growth pattern shows a delay in log phase of resistant bacteria which could be attributed to the necessity of supplementary energy to gain resistance and therefore compensate growth [29]. The results suggest the slow growth of resistant as compensation in order to survive against the bacteriocin by diverting the use of energy in carrying out necessary changes in the cell envelope rather than for growth. The percent cell surface hydrophobicity of resistant strain was found to be higher as compared to wild type strain (Figure 4). Unpaired *t*-test revealed significant differences among wild type and resistant variants (*p* < 0.001). Increased cell surface hydrophobicity may be attributed to the observed higher clumping. The hydrophobic and hydrophilic nature of bacterial cell surface play important role in determining its adherence to living and nonliving surfaces [30].* S. aureus* cell membrane is characteristically hydrophobic in nature [20]. Waters and Dunny [31] have reported that the expression of aggregation substance on the cell surface of* E. faecalis* cells resulted in a significant increase in cell surface hydrophobicity. Surface hydrophobicity of quaternary ammonium compounds (QAC) and amphoteric resistant cells was higher than that of unadapted cells. The levels of resistance increased in a linear way, as the cell surface hydrophobicity of resistant variants increased compared to the cell surface hydrophobicity of wild type strain [15, 32]. Reduced cell surface hydrophobicity was observed in* S. aureus *as an adaptive measure [1, 33]. Teichoic acids of* S. aureus *and other Gram positive bacteria consist of alternating glycerolphosphate or ribitolphosphate units, which are substituted with N-acetylglucosamine or D-alanine [34]. These polymers are anchored to either the cytoplasmic membrane (lipoteichoic acid) or the peptidoglycan (wall teichoic acid) and show anionic properties due to the presence of negatively charged phosphate groups. By substitution of teichoic acids with D-alanine, positive amino groups are introduced, which leads to a partial neutralization of the polymer [35]. The reduced negative charge reduces the possible repulsive forces between staphylococcal bunches and results in larger clumps. As shown in a previous report, nisin-resistant* Streptococcus bovis* cells had more lipoteichoic acid (LTA) than sensitive cells. The primary cell aggregates of resistant bacteria may produce exopolysaccharides to facilitate clumping or aggregation ultimately leading to cell multiplication and formation of a mature structure consisting of many layers of cells [36]. Contradictory to this, reduced cell surface hydrophobicity and increased thickness of the cell wall have been suggested as Gram positive defense mechanisms to limit interactions with lipids [33, 37]. Cell wall composition is also a determinant of cell surface hydrophobicity [38]. Thus, the changes in cell surface hydrophobicity point toward altered cell wall. The results of the present study show that increased hydrophobicity leads to staphylococcal bunches in close proximity and thus to larger bunches, in order to defend against the bacteriocin. The bacterial cell envelope is negatively charged; the increased hydrophobicity and increased clumping suggest a less negative charge which leads to a stable clump formation without any repulsion which interferes with the cationic antimicrobial peptide interaction to the bacterial cell and makes it ineffective or less effective against the resistant.

## Figures and Tables

**Table 1:** 

Strain	IC_50_^*∗*^ (*µ*g/mL)	Reference
*Staphylococcus aureus* NCDC 133		
Wild type	5.5	Lather et al., 2014 [19]
Resistant variant	55.7	Lather et al., 2014 [19]

^*∗*^IC_50_: 50% inhibitory concentration.
